# Precise Dose of Folic Acid Supplementation Is Essential for Embryonic Heart Development in Zebrafish

**DOI:** 10.3390/biology11010028

**Published:** 2021-12-26

**Authors:** Xuhui Han, Bingqi Wang, Dongxu Jin, Kuang Liu, Hongjie Wang, Liangbiao Chen, Yao Zu

**Affiliations:** 1 International Research Center for Marine Biosciences, Ministry of Science and Technology, College of Fisheries and Life Sciences, Shanghai Ocean University, Shanghai 201306, China; m190110412@st.shou.edu.cn (X.H.); m200100094@st.shou.edu.cn (B.W.); m210100123@st.shou.edu.cn (D.J.); m210100127@st.shou.edu.cn (K.L.); m200100110@st.shou.edu.cn (H.W.); lbchen@shou.edu.cn (L.C.); 2 Key Laboratory of Exploration and Utilization of Aquatic Genetic Resources, Ministry of Education, College of Fisheries and Life Sciences, Shanghai Ocean University, Shanghai 201306, China; 3 Key Laboratory of Freshwater Aquatic Genetic Resources, Ministry of Agriculture, College of Fisheries and Life Sciences, Shanghai Ocean University, Shanghai 201306, China

**Keywords:** folic acid, *mthfr*, heart development, zebrafish, CRISPR/Cas9

## Abstract

**Simple Summary:**

Folic acid is an essential vitamin for human beings. It has become a consensus to supplement folic acid during pregnancy. It is reported that 15~20% of people in the world supplement folic acid excessively. We found that excessive folic acid supplementation or insufficient folic acid intake could lead to abnormal heart development in zebrafish embryos. We elucidated the mechanism of folic acid on early cardiac development for the first time. These results provide a scientific basis for the important reasonable supplement dose of folic acid. At the same time, we constructed zebrafish mutants with abnormal folate metabolism, which provide a novel biological model for the study of folate acid metabolism.

**Abstract:**

Folic acid, one of the 13 essential vitamins, plays an important role in cardiovascular development. Mutations in folic acid synthesis gene 5,10-methylenetetrahydrofolate reductase (*MTHFR*) is associated with the occurrence of congenital heart disease. However, the mechanisms underlying the regulation of cardiac development by *mthfr* gene are poorly understood. Here, we exposed zebrafish embryos to excessive folate or folate metabolism inhibitors. Moreover, we established a knock-out mutant of *mthfr* gene in zebrafish by using CRISPR/Cas9. The zebrafish embryos of insufficient or excessive folic acid and *mthfr^−/−^* mutant all gave rise to early pericardial edema and cardiac defect at 3 days post fertilization (dpf). Furthermore, the folic acid treated embryos showed abnormal movement at 5 dpf. The expression levels of cardiac marker genes *hand2*, *gata4,* and *nppa* changed in the abnormality of folate metabolism embryos and *mthfr^−/−^* mutant, and there is evidence that they are related to the change of methylation level caused by the change of folate metabolism. In conclusion, our study provides a novel model for the in-depth study of *MTHFR* gene and folate metabolism. Furthermore, our results reveal that folic acid has a dose-dependent effect on early cardiac development. Precise dosage of folic acid supplementation is crucial for the embryonic development of organisms.

## 1. Introduction

Folic acid plays a vital role in cardiovascular development as it is an important vitamin necessary for methylation reaction, nucleotide synthesis and maintaining homocysteine at a non-toxic level [1]. Insufficient folate metabolism will lead to methionine circulation obstruction and Hyperhomocysteinemia (Hhcy) [2]. Hhcy is an independent risk factor for congenital heart disease (CHD) [3]. Some studies have shown that folic acid deficiency can also affect the development of the brain, liver, and other organs, leading to the occurrence of various diseases [4,5]. In the world, taking folic acid supplementation during pregnancy to prevent the occurrence of various congenital diseases has become a consensus. However, the combined intake of folic acid and folic acid supplements included in the daily diet results in an intake dose of folic acid that exceeds the recommended dose by a factor of 1–4. Thus 15–20% of pregnant women still overdose on folic acid supplements [6,7]. Therefore, it is very important to evaluate the effects of folic acid deficiency and excess on organ development.

Low folate intake during pregnancy and a common genetic variation in folate metabolism (methylenetetrahydrofolate reductase (MTHFR), 677 C > T, about 15% homozygous and 20% heterozygous in Caucasians, about 17% homozygous and 23% heterozygous in Northern Chinese) can lead to varying degrees of the fetal dysplasia [8,9]. MTHFR is one of the key enzymes in folate pathway and methionine metabolism. Under the action of methylenetetrahydrofolate reductase and methionine synthetase reductase (MTRR), folate participates in two important aspects of metabolism in the human body. Folic acid can add the chemical group of “one-carbon unit” to the harmful homocysteine, thus reducing the level of Hcys in plasma [2]. It is very necessary to study the regulation of folic acid in early biological development.

The metabolism of folic acid includes the transformation of exogenous folic acid into 5-methyltetrahydrofolate (5-MTHF) and the methylation of 5-MTHF into Hcys [10,11]. As a carrier of one-carbon unit, folic acid mediates the transfer of one-carbon unit in the form of coenzyme in the process of amino acid metabolism and mutual transformation between methionine and Hcys [12,13]; these reports indicate that folic acid plays an important role in the early development of biological organs, especially in the development of neural tube. However, it is still unclear whether the excessive and deficient folate acid will affect the heart development and the mechanism of folate acid affecting the development of organs including cardiogenesis.

In this study, we used different concentrations of folic acid and folic acid inhibitor methotrexate to treat zebrafish embryos. We aim to explore the effects of excessive or insufficient folic acid supplementation on early heart development of zebrafish embryos. A zebrafish knock-out model of *MTHFR* gene was constructed by CRISPR/Cas9 technology to determine the dose-dependent effect and mechanism of folic acid on embryonic heart development. This study broadens our understanding of the potential function of *MTHFR* gene and deepens the current understanding of the relationship between folate acid and early heart development.

## 2. Materials and Methods

### 2.1. Zebrafish Maintenance and Care

The adult AB wild-type zebrafish maintained at 28.5 °C in the dark cycle of 14 h/10 h light. Five to six pairs of zebrafish mated naturally. An average of 200–300 embryos were produced each time. The feeding and mating spawning plan is carried out according to the description of the zebrafish book [14]. Embryos were stored, washed, and graded in E3 medium (5 mM NaCl, 0.17 mm KCl, 0.33 mm CaCl_2_, 0.33 mm MgSO_4_ dissolved in redistilled water) at 28.5 °C. The imaging experimental embryos which older than 24 h are treated with phenylthiourea (PTU) to prevent the formation of melanin. Use 0.1% DMSO to dissolve folic acid and folic acid antagonist, set different concentration gradients to soak zebrafish in a petri dish for a specific time. All handling of fish was carried out in accordance with the guidelines on the care and use of animals for scientific purposes set up by the Institutional Animal Care and Use Committee (IACUC) of the Shanghai Ocean University (SHOU), Shanghai, China. This research was approved by the IACUC (IACUC 20171009) of SHOU.

### 2.2. Visible Light Imaging and Fluorescence Imaging of Zebrafish Embryos

The zebrafish embryos were placed in MS222 with a concentration of 150 mg/L for 60 s and then anesthetized. When placed in a low-temperature environment, the heart stopped beating. Put them into 1% 2-hydroxyethyl agarose (Sigma-Aldrich, St. Louis, MO, USA, A9414) in the lateral view and ventral view, respectively, to complete the confocal microscope imaging (ZEISS^®^ LSM710, Germany). Fluorescently labeled zebrafish are *cmlc2*:EGFP and *kdrl:mCherry* transgenic fish lines.

### 2.3. Microinjection of Zebrafish Embryos with CRISPR/Cas9 Knock-Out

The gRNA backbone plasmid was obtained from Xiong’ lab [15]. sgRNAs were designed against the *mthfr* gene (ENSDARG00000053087) using the CRISPR design tool (http://crispr.mit.edu/ accessed on 15 November 2020) [16]. The target was designed as Exon2 5′-GGTGAACCAAAGAGCTGACG-3′. The sequence of primers was as follows Primers forward 5′-GGGGTAATGCTGCCAACTGA-3′ Primers reverse 5′-GATTGACCGCTCCAGACGAT-3′. Maxiscriptt7 was used to transcribe in vitro, and then sgRNA was obtained by LiCl/ethanol precipitation. At the single-cell stage, embryos were injected with 1 nL solution containing 80 ng/µL *mthfr*-sgRNAs and 400 ng/µL cas9 protein. Then the injected embryos were cultured in E3 medium.

### 2.4. Protein Structure Prediction

The nucleotide sequences obtained from the mutants’ genome sequencing were translated to protein sequences using the ExPASy-Translate tool. The protein structures were predicted using the Swiss Model Tool (https://www.swissmodel.expasy.org/ accessed on 20 January 2021).

### 2.5. Cardiac Physiology Measurement

We used Zeiss Axioplan microscope (Germany) to record the embryonic cardiac physiology. Place the 3 dpf zebrafish under the microscope and place it in 3% methylcellulose for shooting. Ventricular diastolic area, systolic area, and heart rate were measured using ImageJ (NIH, USA). Zebrafish ventricular diastolic volume (DV) was estimated as DV = 4/3π*L*S^2^ (L and S represent the length of the long axis and short axis, respectively).

### 2.6. In Situ Hybridization

Whole mount in situ hybridization (WISH) was performed as previously described by Tong and Zu et al. [17]. Primers used for antisense probe synthesis were listed in supplementary Table S1.

### 2.7. Quantitative Real-Time PCR

Total RNA was extracted by homogenizing 30 embryos in Trizol reagent (Invitrogen, Waltham, MA, USA, 15596-026), followed by standard reverse transcription. Using SYBR^®^ Green Master Mix (Thermo Fisher, Waltham, MA, USA, A25742) was used for quadruplicate real-time PCR. Primer sequences are listed in supplementary Table S2. β-actin was used to standardize the gene level. The change of multiple was calculated by 2^−^^△△^^Ct^ method. Statistically significant differences were defined as *p* < 0.05 using Student’s *t*-test.

### 2.8. Luciferase Assays

HEK293T cells were plated in 24-well plates and transfected using FuGENE^®^ HD Transfection Reagent (Promega, Madison, WI, USA) with 100 ng reporter. Add 1 mM and 5 mM active folic acid to the cell culture medium. Luciferase activity was measured 36 h after transfection by using the Dual-Luciferase Reporter Assay System (Promega, Madison, WI, USA). At least three independent experiments were performed in triplicate, and statistical significance was calculated with one-way ANOVA test in GraphPad Prism 6.0.

### 2.9. Statistical Analysis

The data derived from more than three independent sets of experiments for qPCR and luciferase assays were evaluated using one-way ANOVA and GraphPad Prism 6.0 for comparison between groups, and *p* ≤ 0.05 was considered to be statistically significant.

### 2.10. Behavioral Test

Behavioral tests were performed at 5 dpf and recorded using the Danio vision system (Noldus information technology company, Wageningen, The Netherlands). Zebrafish were divided into wild type, MTX antagonistic and folic acid excess groups. Embryos were collected and placed in a 24 well E3 medium, one embryo per well. After 15 min of indoor adaptation, 10 min of exercise were recorded. Digital tracks and heat maps were generated using the Ethovision^®^ XT 11.5 software (Noldus). The above measurements are made in triplicate. Mobility refers to the proportion of the area swept by zebrafish swimming in a certain period of time.

### 2.11. S-Adenosylmethionine Content Determination

The zebrafish hearts were peeled off under a microscope, and 30 hearts were peeled off for lysis in each treatment group. Homogenize using a dounce homogenizer and make sure the tissue is lysed. Centrifuge at 13,000× *g* for 15 min at 4 °C. Take the supernatant and filter the samples using a 10 kDa spin column (10,000× *g*, 4 °C, 10 min). Retain the ultra-filtrate for the assay. Prepare the equilibrated standards, controls and samples under room temperature. Set up standard wells, sample wells and sample background control wells. Incubate the plate for 30 min at 37 °C and measure fluorescence at Ex/Em = 535/587 nm in endpoint mode. C_Met = Methionine amount from a standard curve (pmol)/Volume of sample (µL) * Dilution factor.

### 2.12. Assessment of DNA Methylation Level

Bisulfite sequencing PCR (BSP) was used to verify the change of DNA methylation level. Genomic DNA was treated with bisulfite according to the operation manual of EZ DNA methylation gold^®^ Kit (Zymo, Irvine, CA, USA, d5005). Three groups of offspring of the same parents were used in each experiment, and three technical repetitions were completed. The primers of BSP-PCR were designed by methprimer (http://www.urogene.org/cgi-bin/methprimer/methprimer.cgi accessed on 23 May 2021). The primer sequence is in the supplementary table. DNA fragments were cloned and sequenced by trans1-t1 competent cells. BIQ analyzer software was used to analyze the sequencing results.

## 3. Results

### 3.1. Folic Acid and MTX Showed Dose-Dependent Effects on Early Cardiac Development in Zebrafish

To evaluate the insufficient or excessive folic acid effect on cardiac development, we used transgenic zebrafish embryos Tg (*cmlc2:EGFP;kdrl:mCherry*) to investigate the role of exogenous folate in cardiac development after normal neural tube closure. Therefore, the exposure time was 16 hpf to 36 hpf.

**Figure 1:**
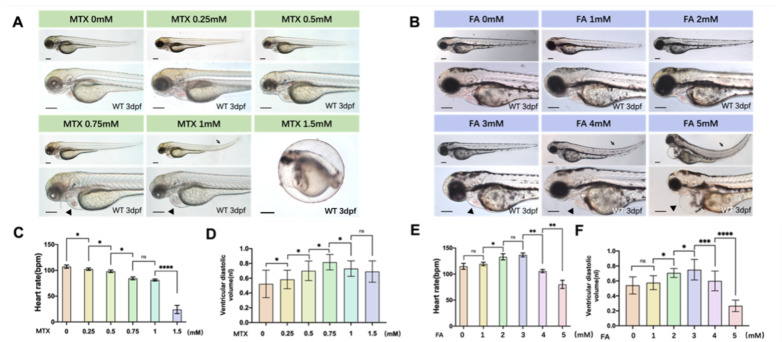
Zebrafish heart dysplasia caused by folic acid and folic acid inhibitors is dose-dependent. (**A**) The use of different concentrations of folic acid inhibitors can cause abnormal heart development in zebrafish embryos. The concentration of MTX1.5 mM will cause the zebrafish embryo to be deformed and unable to break the eggs. Arrowheads mark abnormal heart development. Arrows indicate trunk flexion. Scale bars: 500 μm. (**B**) Excessive folic acid can also cause abnormal heart development in zebrafish embryos. Scale bars: 500 μm. (**C**) The heart rate of zebrafish embryos treated with folic acid inhibitors was statistically analyzed. (**D**) The ventricular diastolic volume of zebrafish embryos treated with folic acid inhibitors was statistically analyzed. (**E**) The heart rate of zebrafish embryos treated with different concentrations of folic acid was analyzed statistically. (**F**) The ventricular diastolic volume of zebrafish embryos treated with different concentrations of folic acid was statistically analyzed. (Student *t*-test, * *p* < 0.05, ** *p* < 0.01, *** *p* < 0.001, **** *p* < 0.0001, ns: no statistical difference).

Embryos were kept in E3 medium and exposed to inhibitors methotrexate (MTX) and different doses of folic acid. We set up ten groups of exposures at different concentrations, named the control group, 0.25 mM, 0.5 mM, 0.75 mM and 1 mM MTX, and recommended [7] doses of folic acid (FA 1 mM) and five-fold excess folic acid (FA 5 mM). We also divided the dosing interval into equal parts to test if there are dose-dependence effects. There are 30 embryos in each group, and 3 biological replicates are set. At 3 dpf, abnormal zebrafish embryonic heart development was observed. With increasing exposure concentrations and abnormal heart development, secondary effects such as trunk flexion occurred later. (Figure 1A,B). As the dose of antagonist and folic acid increased, the heart rate of zebrafish embryos decreased with the use of antagonists (Figure 1C). The ventricular diastolic volume of zebrafish embryos also changed (Figure 1D), which seems to be related to structural abnormalities during heart development. These phenomena were also manifested in the folic acid overdose treatment group. The heart rate of zebrafish embryos increased first and then decreased with increasing concentration of folic acid (Figure 1E). The ventricular diastolic volume also increased first and then decreased (Figure 1F). These results indicate that excessive folic acid supplementation and the use of antagonists can cause abnormal development of zebrafish embryos, and the heart is one of the earliest abnormal developmental organs (Figure S1A,B). These changes are statistically confirmed and these data showed there is a dose-dependent effect of folic acid on zebrafish cardiogenesis.

### 3.2. Insufficient or Excessive Folic Acid Leads to Heart Looping Defect and Abnormal Homocysteine Metabolism

**Figure 2:**
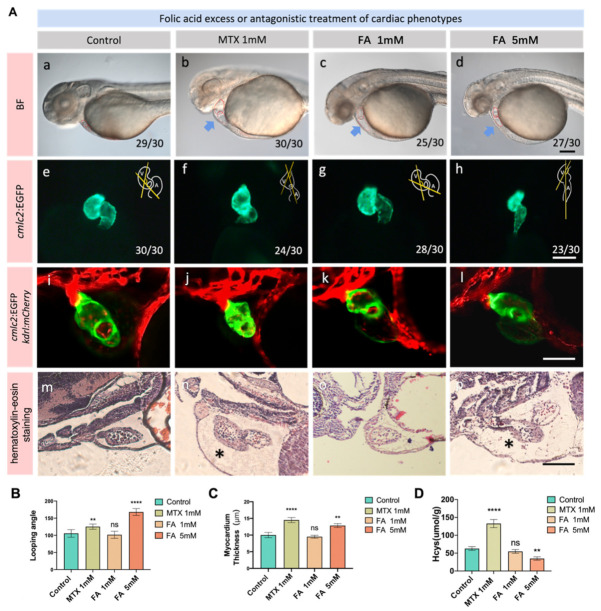
Folate excess and deficiency lead to different degrees of abnormal cardiac physiological function and abnormal metabolism of homocysteine. (**A**) The phenotypes of zebrafish embryonic heart treated with different concentrations of folic acid and MTX inhibited folate metabolism pathway were demonstrated at 3 dpf. (**a**–**d**) is the view under light microscope, (**e**–**h**) is *cmlc2:EGFP* labeled cardiac fluorescence images, (**i**–**l**) is *cmlc2:EGFP* and *kdrl:mCherry* Co labeled confocal images of the heart. (**m**–**p**) is images of a paraffin section of a zebrafish heart after staining by hematoxylin-eosin. Scale bars: 100 μm. (**B**) Statistics on the influence of different folic acid treatment groups on the looping angle of zebrafish embryo heart. (**C**) Statistics on the influence of different folic acid treatment groups on the thickness of the ventricular myocardium of zebrafish embryos. (**D**) High homocysteine content in zebrafish of different groups. (Student *t*-test, * *p* < 0.05, ** *p* < 0.01, **** *p* < 0.0001, ns: no statistical difference).

To investigate how the folic acid metabolism affects embryonic heart development, we used *cmlc2*:EGFP; *kdrl:mCherry* transgenic fish line to trace the development process of the exposed embryos. At 36 hpf, we observed that the heart development first appeared abnormal. Compared with the control group, the ventricle of the folic acid antagonist group tended to expand, the atrium and ventricle of the folic acid overdose group were abnormal, and the heart looping showed abnormal signs (Figure 2A(a–d)). In our previous studies, we have found that folic acid inhibitors can cause significant pericardial swelling and ventricular dilation in zebrafish embryos when they develop to 3 dpf. Further observation of the 3 dpf zebrafish embryonic heart under a fluorescence microscope revealed that folic acid inhibitors can cause abnormal heart looping. Excessive folic acid stretched the atrium and ventricle of zebrafish embryos and cause abnormal cardiac circulation (Figure 2A(e–h)). These results indicate that both folic acid deficiency and excess can cause abnormal heart development in zebrafish embryos. Confocal images showed abnormal heart looping after folic acid overdose and MTX folic acid antagonist treatment in zebrafish. At the same time, the structure of the heart chamber had also changed, and the thickness of the myocardial wall increased with the exposure dose (Figure 2A(i–l)). Zebrafish embryo slice images exposed to different key concentrations of folic acid excess or inhibition groups reflected the thickness changes of the myocardium (Figure 2A(m–p)). According to the statistics of cardiac physiological data, excessive folate showed a more serious abnormal effect on heart looping (Figure 2B). Compared with excessive folate, folic acid antagonism increased the thickness of the ventricular myocardial wall severer (Figure 2C).

In order to find the reason for this phenomenon, we tested the concentration of the homocysteine, which is known in the folic acid metabolism pathway and also related to the abnormal development of the heart. It was found that the different intake doses of folic acid caused significant changes in the content of homocysteine in zebrafish embryos (Figure 2D). Hyperhomocysteinemia is an independent risk factor for congenital heart disease, and zebrafish embryonic heart development is sensitive to changes in homocysteine.

### 3.3. Abnormal Folate Metabolism Leads to Behavior Disorder of Zebrafish Embryos

**Figure 3:**
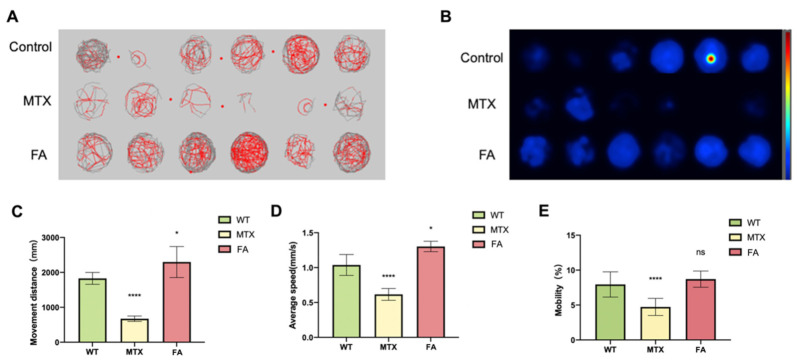
Zebrafish Behavioral Experimental Data Statistics. (**A**) Digital tracks of larvae from wildtype (WT), MTX 1 mM and FA 1 mM groups at 5 dpf. (**B**) Heat maps of the digital tracks A. (**C**) The ten-minute activity distance statistics of zebrafish embryos in different groups. (**D**) Statistics on the average activity speed of zebrafish embryos in different groups. (**E**) Mobility statistics of zebrafish embryos in different groups. (Student *t*-test, * *p* < 0.05, **** *p* < 0.0001, ns: no statistical difference.).

Excessive homocysteine content can cause fatigue and slow movement in mammals. After we discovered that abnormal folic acid intake in zebrafish caused an increase in homocysteine content, we conducted zebrafish behavioral experiments. We observed abnormal behavior changes in zebrafish treated with folic acid or folic acid antagonists. Behavioral testing was conducted at 5 dpf. The digital trajectory and the corresponding heat map were shown in (Figure 3A,B). Compared with wild-type embryos, the movement distance and average speed of zebrafish embryos in different exposure groups were observed (Figure 3C,D). Mobility was the percentage of the completed area (Figure 3E). Super excessive folic acid supplementation caused zebrafish embryos to die within 5 dpf, and no statistics on behavioral data were available. The blocked folate cycle caused the zebrafish embryos to reduce their exercise capacity. Zebrafish exhibit reduced exercise status similar to human hyperhomocysteinemia. Different concentrations of folic acid were used in HEK293T cells and detected by the luciferase reporter gene system. We found that changes in folate content can also cause changes in some genes related to the regulation of heart development (Figure S2).

### 3.4. CRISPR/Cas9 Mediated mthfr Gene Knock-Out Model in Zebrafish

**Figure 4:**
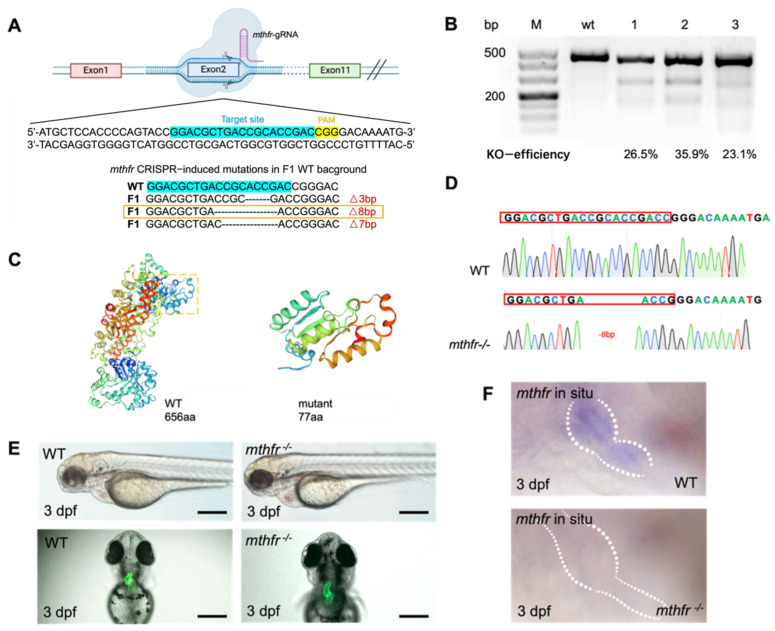
Generation of *mthfr* mutant using the CRISPR/Cas9 system. (**A**) Design of sgRNA target on exon 2 of zebrafish *mthfr* gene. The mutation type of code shift selected by orange frame is selected finally. (**B**) Two short DNA fragments were obtained by T7E1 digestion. In the control group, 437 bp fragment was amplified from wild-type embryos with the same set of primers. (**C**) The predicted truncation of Mthfr protein. (**D**) Sequencing map of homozygous zebrafish adults. (**E**) Bright-field views of *mthfr^−/−^* zebrafish showed Pericardial enlargement, ventricular enlargement and abnormal heart looping. Scale bars: 500 μm. (**F**) The results of in situ hybridization showed that the expression of *mthfr* gene in homozygous mutant zebrafish was changed.

We constructed the *mthfr* gene knock-out mutant by using CRISPR/Cas9 in zebrafish. *mthfr* gene was knocked out by a specific sgRNA target on exon 2 (Figure 4A). Microinjection of *mthfr* sgRNA and Cas9 protein into the fertilized single-cell embryos were performed. T7E1 assay was performed at 2 days after fertilization to verify the indels of exon 2 of *mthfr* transcript in the injected embryos. The gel results of T7E1 showed that the injected embryos occurred indels on the target exon 2. One fragment was amplified with primers spanning the exon 2 gRNA sequence of *mthfr*, and two shorter DNA fragments were obtained by T7E1 digestion. In the control group, a 437 bp fragment was amplified from wild-type embryos (Figure 4B). Sequencing of PCR products confirmed that the exon 2 of *mthfr* knock-out injected embryo transcript had several base pairs deletion. The genome-editing efficiency of several *mthfr* sgRNA targeting different sites were compared, and one with the highest efficiency for exon 2 was selected. The 8 bp frameshift deletion produced a premature stop codon, which truncated the protein of 77 amino acids (aa) compared with 656aa full-length Mthfr protein (Figure 4C). The *mthfr ^−/−^* fish carrying 8 bp deletion was incrossed to obtain homozygous *mthfr* fish whose genotype was validated by sequencing (Figure 4D). Compared with wild-type zebrafish embryos, *mthfr ^−/−^* mutant zebrafish embryos showed abnormal cardiac development. Pericardial edema, and abnormal heart looping were found (Figure 4E).

In situ hybridization showed that *mthfr* gene was expressed at 3 dpf in the wild-type zebrafish heart. The expression of *mthfr* gene decreased significantly in *mthfr ^−/−^* mutant. (Figure 4F). qPCR experiment showed that the expression of *mthfr* gene increased at 6 hpf, then decreased and stabilized at 12 hpf. After 16 hpf neural tube closure [18], qPCR results showed that *mthfr* gene is still involved in the later organ development (Figure S3A). In situ hybridization confirmed that *mthfr* was widely expressed in zebrafish embryos, including heart, brain, optic nerve and liver. It is strongly expressed in the heart (Figure S3B). These results indicate that *mthfr* gene is involved in folate metabolism and plays an important role in the early embryonic development of zebrafish.

### 3.5. Folic Acid Deficiency and Excessive Supplement Lead to the Changes of Heart Related Genes

**Figure 5:**
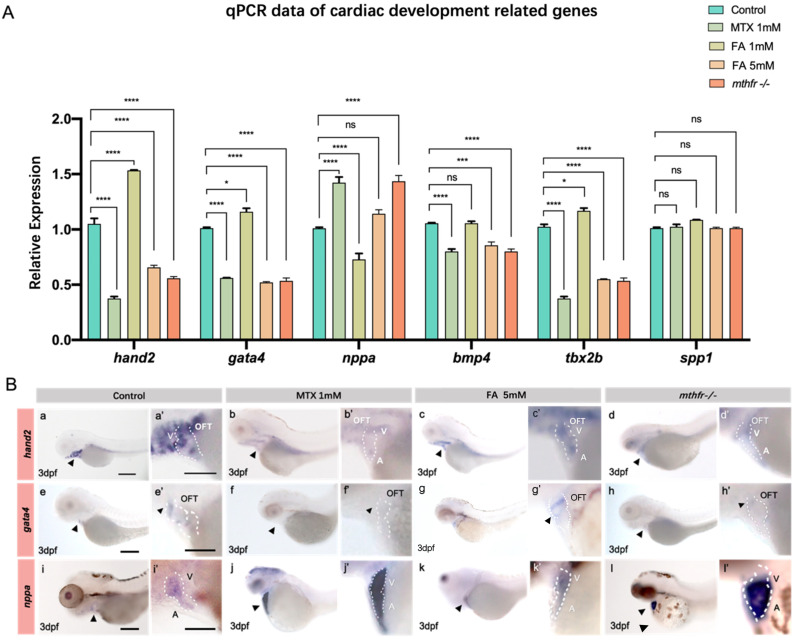
Abnormal folate metabolism results in changes of zebrafish embryo heart development gene expression. (**A**) Results of qPCR for *hand2, nkx2.5, gata4, bmp4, tbx2b, spp1, hoxb1a, nppa* in the embryonic heart of zebrafish in each group. (Student *t*-test, * *p* < 0.05, *** *p* < 0.001, **** *p* < 0.0001, ns: no statistical difference.) (**B**) In situ hybridization showed the expression of *hand2*, *gata4*, *nppa* in different groups of 3 dpf zebrafish embryonic hearts. (**a**–**d‘**) is the in situ expression of the *hand2* gene at different exposure concentrations, (**e**–**h‘**) is the in situ expression of the *gata4* gene at different exposure concentrations, (**i**–**l‘**) is the in situ expression of the *nppa* gene at different exposure concentrations. A: atrium V: ventricle OFT: outflow tract. *n* = 3. Scale bars: 200 μm.

The expressions of *hand2, gata4, nppa, bmp4, tbx2b, spp1* and et al. in the heart of zebrafish embryos at 3 dpf were detected by qPCR. Compared with the untreated control group, the changes of folate supplement and the expression of genes related to heart development in *mthfr ^−/−^* mutant embryos were different (Figure 5A).

According to the results of qPCR and the previous abnormal phenotype of the heart, *hand2, gata4* and *nppa* were selected for the further in situ hybridization experiments. In wild-type zebrafish embryos, *hand2* was expressed in the AVC (A-V canal) of the heart at 3 dpf (Figure 5B(a)). The expression of *hand2* in zebrafish embryonic heart was decreased in folic acid antagonistic treatment group, folic acid excess supplement group and *mthfr ^−/−^* mutation group (Figure 5B(b–d)). In wild-type zebrafish embryos, *gata4* was strongly expressed in cardiac outflow tract (OFT) and AVC (Figure 5B(e)). Similarly, the expression of *gata4* gene decreased with the metabolism of folic acid (Figure 5B(f–h)). This is consistent with the qPCR expression trend of *hand2*. In the zebrafish embryonic heart, *nppa* gene expression was up-regulated with the decrease of folate metabolism (Figure 5B(i–l)).

### 3.6. Folate Metabolism Changes Defect the Methylation of hand2

**Figure 6:**
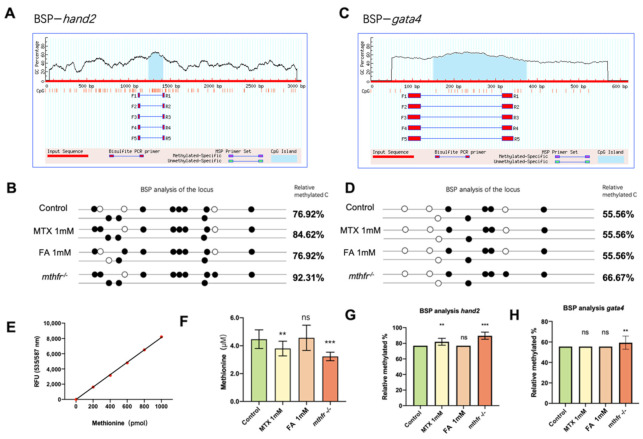
Effects of folic acid on partial gene methylation in zebrafish embryos. (**A**) The CpG island of *hand2* gene for the BSP experiment was predicted and selected. (**B**) Zebrafish embryos with different folate metabolism groups BSP analysis of the locus of *hand2*. (**C**) The CpG island of *gata4* gene for the BSP experiment was predicted and selected. (**D**) Zebrafish embryos with different folate metabolism groups BSP analysis of the locus of *gata4*. (**E**) Standard curve fitting based on the standard sample. (**F**) Methionine levels in heart cells of different folate treatment groups and mthfr homozygous mutants. (**G**) Relative methylated BSP analysis of the *hand2* promoter locus. (**H**) Relative methylated BSP analysis of the *gata4* promoter locus. (Student *t*-test, ** *p* < 0.01, *** *p* < 0.001, ns: no statistical difference).

Epigenetic regulation of gene expression involves many processes [19]. We used bisulfite sequencing (BSP) to verify that abnormal folate metabolism can change the methylation level of *hand2* gene. BSP primer sequence was designed by predicting the CpG island of *hand2* gene promoter (Figure 6A) and the distance between the promoter region and the first exon is 2067 bp. The results showed that folate inhibition and abnormal folate metabolism caused by *mthfr ^−/−^* mutation could increase the methylation level of *hand2* gene (Figure 6B,G). The increase of promoter methylation level can inhibit the expression of *hand2* in zebrafish early embryonic development. Compared with the effect of folic acid on the methylation level of *hand2* gene, the change of folic acid content had no significant effect on the methylation level of *gata4* gene. Only in *mthfr ^−/−^* mutant, the methylation level of *gata4* gene was slightly up-regulated (Figure 6D,H).

SAM is an important donor for DNA methylation. We measured the levels of methionine in the hearts of different groups with abnormal folic acid intake and abnormal metabolism. We found that insufficient folate metabolism can lead to decreased methionine levels in zebrafish heart cells, both in MTX inhibition and *mthfr ^−/−^* mutants (Figure 6F). Appropriate supplementation of folic acid did not significantly change the degree of methylation of zebrafish embryonic heart cells. Therefore, donor changes may be a key factor affecting DNA methylation.

## 4. Discussion

This study found that excessive or insufficient folate can lead to abnormal heart development, and *mthfr* gene is one of the key genes. We selected 16 hpf as the initial period of folic acid or MTX exposure. At about 16 hpf, the neural tube of zebrafish closed normally [20]. At 24 hpf, the original cardiac tube was formed by the differentiation of cardiac cells. At 36 hpf, the differentiation of atrium and ventricle was basically completed, and cardiac morphogenesis and torsion completed the development of cardiac looping [21,22]. In this study, we found that after 72 hpf, the abnormal phenotype of the heart caused by folic acid or inhibitor exposure tends to stabilize. Although it has been reported that *MTHFR* gene is closely related to folate metabolism, DNA synthesis and methylation [23], and it has been recognized that folic acid supplementation during pregnancy is beneficial to fetal development, but the mechanism of *MTHFR* gene mutation leading to cardiac dysplasia and increased risk of congenital heart disease is still unclear. We have constructed a zebrafish strain with *mthfr* knock-out, which provides a novel model for exploring the function of *mthfr* in the development of various organs, folate metabolism and DNA methylation of zebrafish early embryos. When zebrafish were severely exposed to excessive folate or MTX, we found that the trunk of zebrafish embryos bent upward. This suggests that when the folate metabolism in zebrafish is seriously abnormal, it may affect the related genes of trunk development [24]. However, abnormal cardiac development occurs earlier than trunk bending.

The development of heart is a complex process, many factors participate in the regulation of heart development. The disorder of these factors can cause the abnormal development of the heart. *hand2*, *gata4*, *nppa, bmp4*, *tbx2b*, *spp1* genes expressed in the heart field, cardiac chamber and atrioventricular septum, which are very important for early cardiac development [25]. Sun et al. [26] found that zebrafish embryos exposed to MTX had abnormal heart development. Additionally, they found that *dhfr-gfp* mRNA or folic acid can improve the heart and blood vessel development of embryos treated with MTX, which proves that MTX inhibits *dhfr*. We found abnormal expression of the *hand2* gene in the zebrafish model with abnormal expression of *mthfr*. This further proves that the expression changes of *hand2* gene are caused by abnormal folate metabolism, rather than simply the inhibition of *dhtr* gene expression. Sun et al. [27] reported that abnormal expression of *dhfr*, a key gene of folate metabolism, and abnormal expression of *hand2*, a key gene of heart development, can lead to abnormal heart development in zebrafish. In our study, we found a correlation between abnormal folate metabolism and abnormal *hand2* expression based on methylation changes. Previously, Yelon et al. [28] reported that the expression of *hand2* had been observed before 24 hpf in zebrafish embryos, and that *hand2* played an important role in the heart precursor from the early stage. Through in situ hybridization experiments, we found that changes in folic acid intake did not seem to significantly change the early expression of *hand2* (Figure S4). Early embryonic development is affected by the interaction of multiple complex pathways, and the specific effects of folic acid on the middle and late stages of embryonic development seem to be more significant. In this study, the expression of *hand2*, *gata4*, *bmp4*, *tbx2b*, *nppa* showed a dose-dependent change of folic acid. Except for *nppa*, the changing trend of these gene expressions is consistent. In addition, excessive folate treatment and folate inhibitor treatment led to down-regulation of gene expression. The results of the in situ hybridization experiments were consistent with the qPCR results. This proves that folate excess and deficiency have a dose-dependent relationship with some genes related to heart development and folate metabolism.

In addition, from the previous Hcys results and behavioral experiments, we found that the increase of exogenous folate could make zebrafish more active. Folic acid deficiency could lead to the increase of Hcys. Interestingly, excessive folate supplementation also led to similar phenotypes and gene expression changes in zebrafish embryos as folate deficiency. Previous studies have shown that folic acid is converted into 5-methyltetrahydrofolate (5-MTHF) in vivo and 5-MTHF enters the methylation reaction of Hcys [29]. Hhcy is an independent risk factor for cardiovascular disease. Hhcy can lead to fatigue, emotional changes and reduced exercise status [30]. However, excessive folic acid did not increase Hcys. These results suggest that folic acid supplementation can lead to changes in the expression of genes related to cardiac development, but it does not affect the cycle of one-carbon unit and the transformation process of folic acid to active folic acid. Studies have shown that the combined effect of genetic and environmental factors may be the main cause of congenital heart disease [22].

Folic acid acts as the carrier of the one-carbon unit in the process of amino acid metabolism and the mutual transformation of methionine and Hcys and mediates the transfer of one-carbon unit in the form of coenzyme. S-adenosylmethionine (SAM) involved in the folate cycle, will also be generated during the folate cycle. As SAM is the most common methyl donor of DNA methylation, it can directly affect the degree of DNA and protein methylation, thus causing apparent genetic disorder [31]. In this study, we determined that folic acid deficiency could increase the methylation level of *hand2* gene promoter. The addition of folic acid can keep DNA methylation at a stable level. Interestingly, we found that the decrease and increase of methionine in the zebrafish heart will cause hypermethylation of some regulatory genes in heart development, and it seems that there is no phenomenon of undermethylation. DNA methylation is affected by many factors. Although the influence of SAM plays a key role in the regulation of DNA methylation, it is difficult to conclude that changes in the content of SAM can be used as all factors for changes in the degree of methylation. Schober et al. [32] reported that the one-carbon metabolite and methyl group donor SAM is pivotal for energy metabolism. This further illustrates that the mechanism by which SAM affects DNA methylation changes is complex and diverse. Although SAM is an important donor of methylation, there may be a unique mechanism for how the metabolism of methionine affects the degree of methylation. Fluorescence images showed that severe overexposure of folic acid would also lead to decreased *kdrl: mCherry* fluorescence and abnormal blood vessel development (Figure S5). This is consistent with the previously reported results [28]. However, while the lowest MTX concentration caused cardiac dysplasia, at this dose the blood vessels did not show any obvious phenotype. After increasing the exposure concentration, abnormal blood vessel development occurred. Fluorescence images of zebrafish embryos showed that early blood vessel development was affected by excessive exposure to MTX or folic acid, which resulted in abnormalities. MTX inhibits the development of intersegmental vessels (ISV) to a certain extent. Excessive folic acid will cause the development of zebrafish dorsal longitudinal anastomotic vessels (DLAV) disorder. It may because DNA synthesis changes before purine synthesis and other protein syntheses. Lee et al. [33] reported that in rapidly dividing hematopoietic or embryonic cells, DNA synthesis is readily compromised prior to purine synthesis and other cellular methylation reactions. Low folate status is considered a potentially reversible risk factor for cardiovascular disease. This has been confirmed in this study, and we also believe that the effect of abnormal folate metabolism on methylation is widespread.

**Figure 7:**
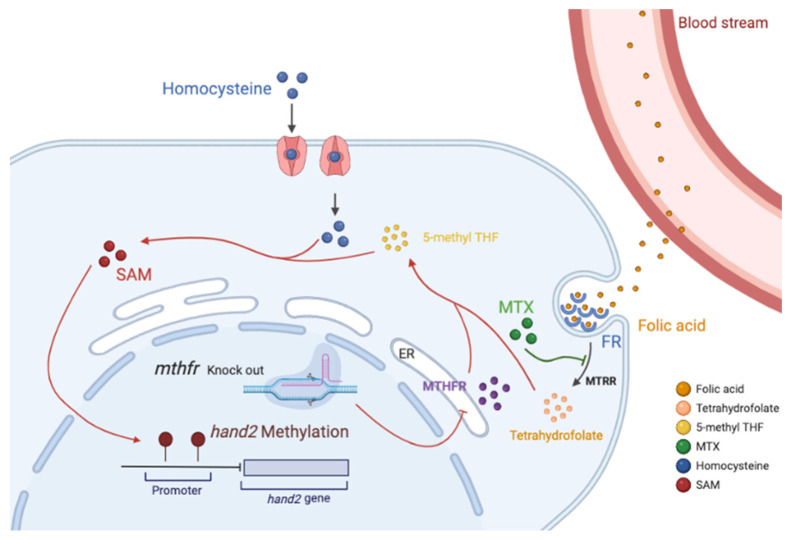
Model of folate dose-dependent regulation on zebrafish heart development. ER: Endoplasmic reticulum; FR: Folate receptor; SAM: S-adenosylmethionine; Folic acid enters the cell through the folate receptor and undergoes the metabolic cycle. MTX specifically antagonizes MTRR, and knockout of the *mthfr* gene inhibits the endoplasmic reticulum synthesis MTHFR enzyme. These factors cause the conversion of folic acid to 5-methyl THF to be blocked, which reduces the efficiency of the conversion of homocysteine to methionine in the cell. Decreasing the content of methionine leads to a decrease in the content of DNA methylation donor SAM, which in turn affects the methylation of the *hand2* promoter. This can lead to abnormal embryonic heart development.

Overall, we constructed a novel *mthfr* zebrafish mutation model and found the first time that insufficient or excessive folic acid intake can lead to changes in gene expression during early cardiac development of zebrafish embryos. Abnormal folate metabolism can lead to abnormal metabolism of Hcys and abnormal methylation of some genes in zebrafish. These results will increase the risk of all kinds of congenital diseases, including congenital heart disease (Figure 7). In contrast, qPCR results and in situ hybridization data revealed that the expression of *mthfr* gene, *hand2* and other cardiac development-related genes in WT zebrafish embryo were more stable and balanced due to the normalization of folate cycle. The homocysteine level in zebrafish tends to be normal. Therefore, the heart development of zebrafish embryos tends to be normal. This also explains why abnormal folate metabolism leads to decreased heart rate and increased signs of heart failure. It has been proved that excessive folic acid supplementation has a negative effect on the early development of organisms. Folic acid has a dose-dependent effect on organism development.

## 5. Conclusions

Folic acid exerts dose-dependent effects on early heart development in zebrafish. Abnormal folate metabolism changes the expression of cardiac development-related genes and the methylation level of *hand2* promoters.

## Data Availability

All data needed to evaluate the conclusions in the paper are included in the paper and/or the Supplementary Materials. The plasmid system and fish lines used in this study are available from the corresponding author upon reasonable request.

## Supplementary Materials

The following are available online at https://www.mdpi.com/article/10.3390/biology11010028/s1, Figure S1: Folic acid antagonists can cause abnormal embryonic development in zebrafish, and the heart is one of the first organs to experience abnormal development, Figure S2: Different concentrations of folate were used in HEK293T cells and detected by luciferase reporter gene system, Figure S3: mthfr gene is widely expressed in zebrafish embryos and qPCR data at different developmental stages, Figure S4: In situ hybridization results showed that the expression of hand2 gene was not sensitive to folate antagonists 24 h ago, Figure S5: Fluorescence images of zebrafish embryos showed that early blood vessel development was affected by excessive exposure to MTX or folic acid, which resulted in abnormalities, Table S1: Primers used for antisense probe synthesis, Table S2: Primers that were used for Quantitative Real-time Polymerase Chain Reaction Video S1: WT and *mthfr* homozygous mutant zebrafish phenotype video.
