# Supplementary material for: Precise Dose of Folic Acid Supplementation Is Essential for Embryonic Heart Development in Zebrafish
Source: Biology (Basel). 2021 Dec 26;11(1):28. doi: 10.3390/biology11010028 (PMC8773176; doi:10.3390/biology11010028)
Supplement: Supplementary file 1 [file biology-11-00028-s001.zip › Supplementary data Han biolgy-1395588-21-12-23 final.pdf]

Supplementary Information

Precise Dose of Folic Acid Supplementation Is Essential for Embryonic Heart Development in Zebrafish

Xuhui Han[1,2,3], Bingqi Wang[1,2,3], Dongxu Jin[1,2,3], Kuang Liu[1,2,3], Hongjie Wang[1,2,3], Liangbiao Chen [1,2,3] , Yao Zu [1,2,3,*]

1. International Research Center for Marine Biosciences, Ministry of Science and Technology, College of Fisheries and Life Sciences, Shanghai Ocean University, Shanghai, 201306, China.

2. Key Laboratory of Exploration and Utilization of Aquatic Genetic Resources, Ministry of Education, College of Fisheries and Life Sciences, Shanghai Ocean University, Shanghai, 201306, China.

3. Key Laboratory of Freshwater Aquatic Genetic Resources, Ministry of Agriculture, College of Fisheries and Life Sciences, Shanghai Ocean University, Shanghai, 201306, China.

* Corresponding author: Yao Zu (Email: yzu@shou.edu.cn) College of Fisheries and Life Sciences, Shanghai Ocean University, Shanghai, 201306, China

Table S1 Primers used for antisense probe synthesis

hand2-probe-F	ACCATGGCACCTTCGTACAG
hand2-probe-R	TAATACGACTCACTATAGGGAGATGGCCAACCAGTTCTCCCTTT
gata4-probe-F	GGTTACCCGGCCTATGTGAG
gata4-probe-R	TAATACGACTCACTATAGGGAGACGCGTCTCTTCTGTTGGACT
nppa-probe-F	TAAGGGGGTCCGAGATCAGG
nppa-probe-R	TAATACGACTCACTATAGGGAGATGCTTCGGGTCGACAATAGG
mthfr-probe-F	ACGTGGGTTGGCACTCTAAG
mthfr-probe-R	TAATACGACTCACTATAGGGAGAAGCTGGGTGACGATGAAGTG

The line is labeled T7 promoter sequence.

Table S2 Primers that were used for Quantitative Real-time Polymerase Chain Reaction

Gene	Forward primer	Revers primer
hand2	GACGCCAAAGAAGAAAGGCG	TCAGCTCCAATGCCCAAACA
gata4	CGCACTTCGACAGCTCCGTACT	AGTCCACAGGCATTGCACAGGTAA
bmp4	AGCCGATCATCTCAACTCCACCAA	TCAGCACCACCCTGTCCGTTTC
tbx2b	GCGAACCAACAACTGGCAGAGATG	GTCCTCACTCCTCGACCTGAATGG
spp1	TCATTCCAGCGACACAACCA	GGCCTGAAATTTGTGGCGTT
nppa	ATTATGAAGACAGCAACACC	GTCAAATCCATCCGAGGG
β-actin	TTCCTTCCTGGGTATGGAATC	GCACTGTGTTGGCATACAGG

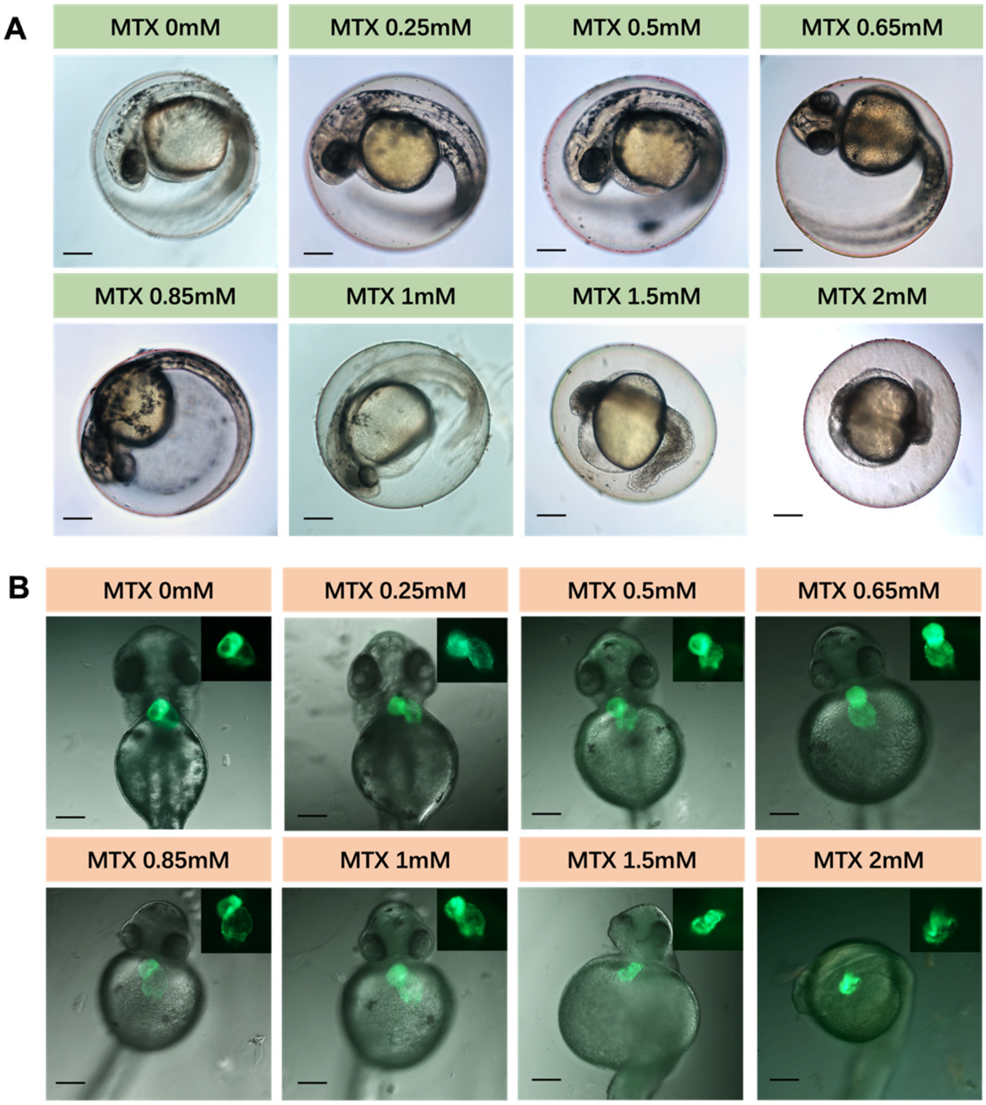

Figure S1 Folic acid antagonists can cause abnormal embryonic development in zebrafish, and the heart is one of the first organs to experience abnormal development. （A）Light microscope photos of 36hpf zebrafish exposed to different MTX concentrations. （B）36hpf zebrafish heart fluorescent label photos exposed to different MTX concentrations, the zebrafish embryos in this group have been stripped of their egg membranes. Scale bars:500μm.

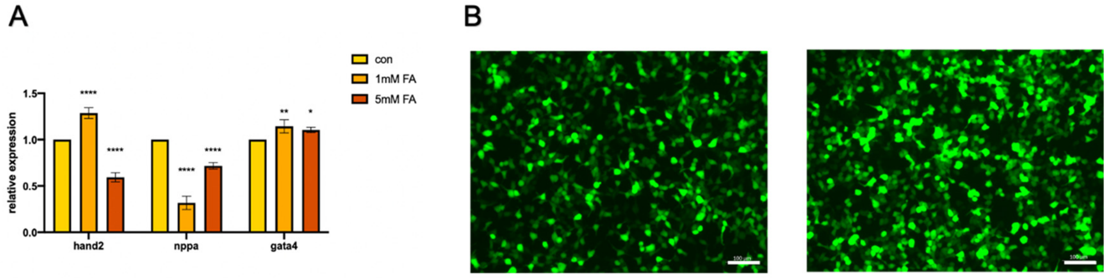

Figure S2 Different concentrations of folate were used in HEK293T cells and detected by luciferase reporter gene system. (A) The expression of *HAND2*, *NPPA* and *GATA4* genes showed dose-dependent changes, which did not change unidirectionally with the change of folate concentration. (B) Green fluorescence plasmid (GFP) was used to determine the transfection efficiency. The figure on the left shows the group with added FA 1mM, and the figure on the left shows the group with added FA 5mM. Scale bars: 100μm.

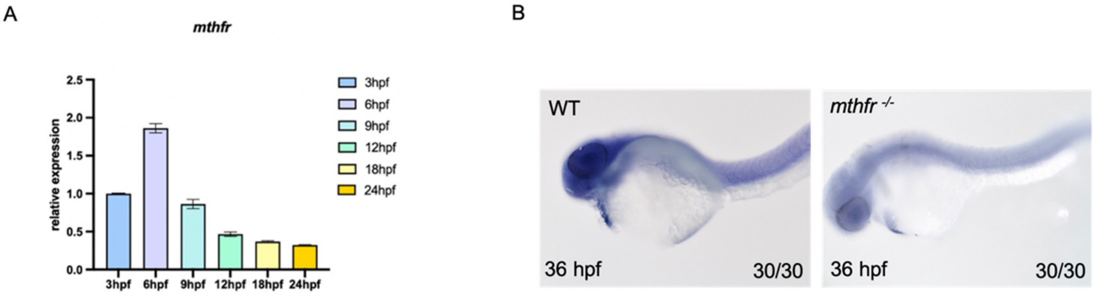

Figure S3 *mthfr* gene is widely expressed in zebrafish embryos and qPCR data at different developmental stages. (A) qPCR results showed that *mthfr* gene was continuously expressed during the early embryonic development of zebrafish. Strong expression was observed at 6hpf, and then decreased. After 12hpf, the expression intensity tended to be stable. (B) The results of 36 hpf zebrafish embryos in situ hybridization showed that *mthfr* gene was widely expressed. The expression signal decreased in *mthfr* mutant.

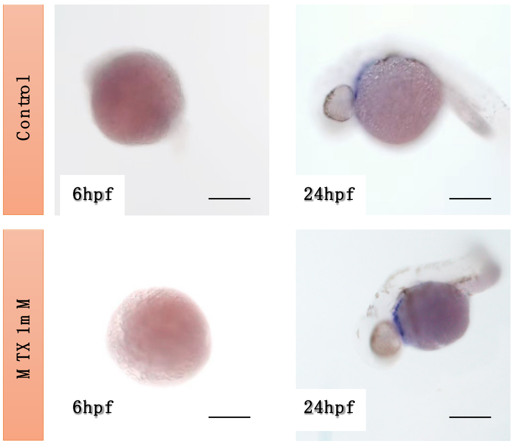

Figure S4 In situ hybridization results showed that the expression of *hand2* gene was not sensitive to folate antagonists 24 hours ago. bars:500μm.

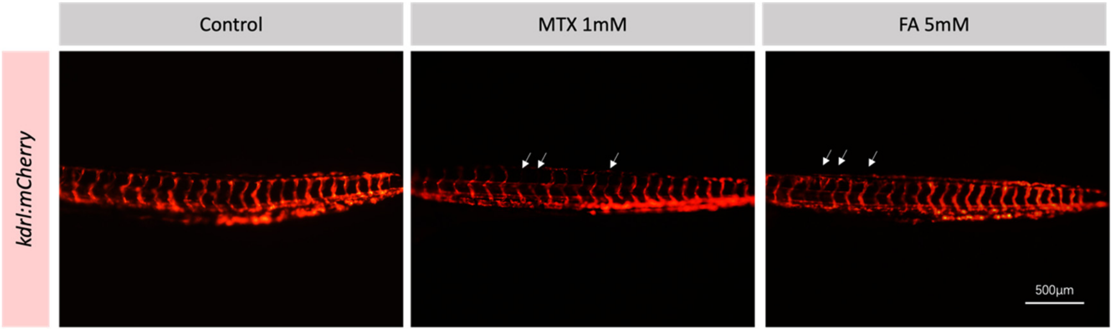

Figure S5 Fluorescence images of zebrafish embryos showed that early blood vessel development was affected by excessive exposure to MTX or folic acid, which resulted in abnormalities. MTX inhibits the development of intersegmental vessels (ISV) to a certain extent. Excessive folic acid will cause the development of zebrafish dorsal longitudinal anastomotic vessels (DLAV) disorder.
