# Supplementary figures and images for: Precise Dose of Folic Acid Supplementation Is Essential for Embryonic Heart Development in Zebrafish
Source: Biology (Basel). 2021 Dec 26;11(1):28. doi: 10.3390/biology11010028 (PMC8773176; doi:10.3390/biology11010028)

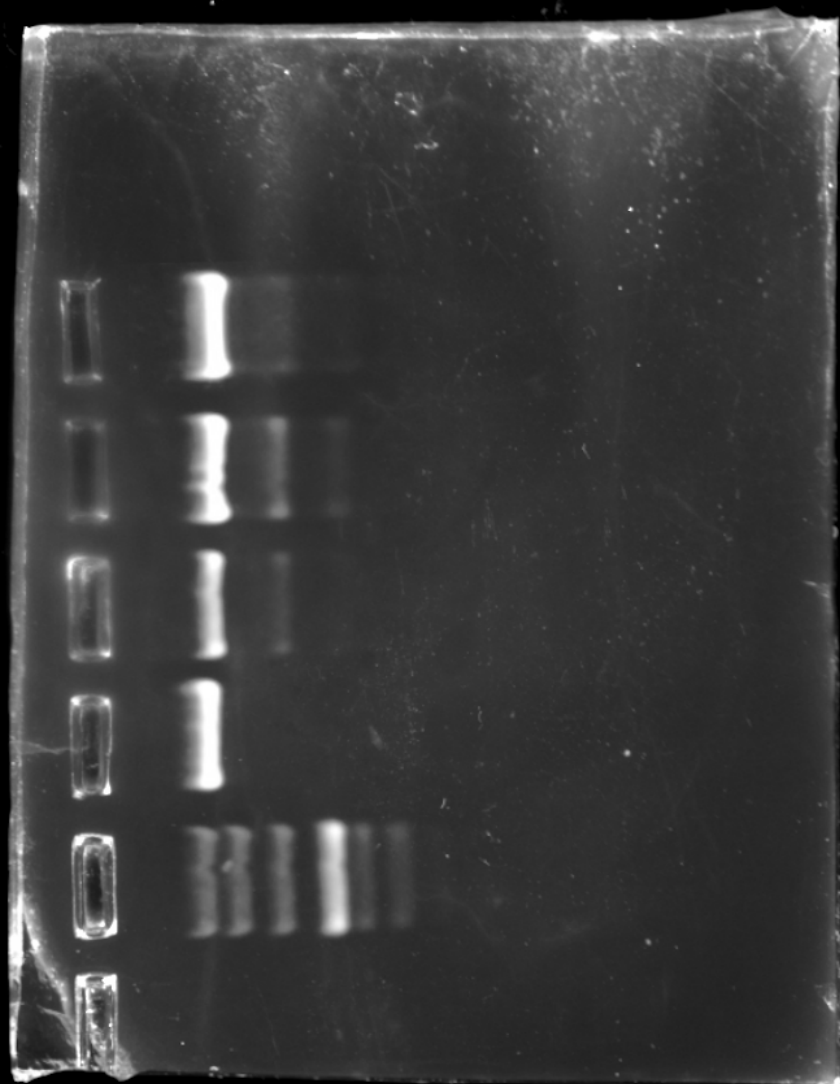

Supplement: Supplementary file 1 [file biology-11-00028-s001.zip › biology-1395588-orm.pdf]
